# Prevalence and factors associated with depression, anxiety, and stress symptoms among home isolated COVID-19 patients in Western Nepal

**DOI:** 10.1016/j.dialog.2022.100090

**Published:** 2022-12-05

**Authors:** Bikram Adhikari, Lisasha Poudel, Tek Bahadur Thapa, Deekshya Neupane, Pranita Maharjan, Ashley Hagaman, Niroj Bhandari, Nishan Katuwal, Bhawana Shrestha, Rashmi Maharjan, Sudip Shrestha, Akina Shrestha, Dipesh Tamrakar, Bibek Rajbhandari, Brish Bahadur Shahi, Rajeev Shrestha, Biraj Man Karmacharya, Archana Shrestha

**Affiliations:** a Research and Development Division, Dhulikhel Hospital, Kathmandu University Hospital, Dhulikhel, Nepal; b Social and Behavioral Sciences Department, Yale School of Public Health, New Haven, USA; c Center for Methods in Implementation and Prevention Science, Yale School of Public Health, New Haven, CT, USA; d Institute for Implementation Science and Health, Nepal; e Department of Community Programs, Dhulikhel Hospital-Kathmandu University Hospital, Dhulikhel, Nepal; f Department of Nursing and Midwifery, Kathmandu University School of Medical Sciences, Dhulikhel, Nepal; g Department of Public Health, Kathmandu University School of Medical Sciences, Dhulikhel, Nepal; h Department of Emergency Medicine and General Practice, Nepal police Hospital, Kathmandu, Nepal; i Ministry of Social Development, Karnali Province, Nepal; j Department of Pharmacology, Kathmandu University School of Medical Sciences, Dhulikhel, Nepal; k Department of Chronic Disease Epidemiology, Yale School of Public Health, New Haven, USA

**Keywords:** COVID-19, Depression symptoms, Anxiety symptoms, Stress symptoms, Home isolation, Nepal

## Abstract

**Introduction:**

Globally, COVID-19 pandemic has a significant impact on mental health. In Nepal, COVID-19 positive cases have to self-isolate at home in multi-generational and multi-family households. This could be strongly associated with depression, anxiety, and stress-related health outcomes. Additionally, COVID-19 related stigma and fear of transmission may intensify depression, anxiety, and stress symptoms. This study determined the prevalence of depression, anxiety, and stress symptoms and their association with presence of COVID-19 symptoms and comorbid conditions among home isolated COVID-19 positives in the Karnali province, Nepal.

**Methods:**

We conducted a cross-sectional study to assess depression, anxiety, and stress symptoms among 402 home isolated COVID-19 patients of Karnali province from January to May 2021 using “Depression, Anxiety and Stress Scale-21 (DASS-21)”. We interviewed patients to collect socio-demographic, DASS-21, COVID-19 symptoms, comorbid conditions, and self-treatment. We conducted a telephonic interview using a standardized questionnaire using Kobotoolbox. We calculated the prevalence of depression, anxiety, and stress symptoms. We utilized univariate and multivariate logistic regression to determine their association with the presence of COVID-19 symptoms and comorbid conditions. In multivariate logistic regression, we adjusted sociodemographic factors (age, gender, ethnicity, marital status, monthly family income, education level), smoking status and history of self-treatment. We reported adjusted odds ratios (aOR) with 95% confidence intervals. All analyses were conducted in R (version: 4.0.3).

**Results:**

The prevalence of depression, anxiety and stress symptoms among home isolated COVID-19 patients were 8.0% (95% CI: 5.5 to 11.1), 11.2% (95% CI: 8.3 to 14.7), and 4.0% (95% CI: 2.3 to 6.4) respectively. Higher odds of depression symptoms (aOR: 2.86; 95% CI: 1.10–7.44, p = 0.03), anxiety symptoms (aOR: 3.81; 95% CI: 1.62 to 8.93; p = <0.01) and stress symptoms (aOR: 7.78; 95% CI: 1.43 to 42.28; p = 0.02) were associated significantly with the presence of COVID-19 symptoms in past week. Higher odds of anxiety symptoms were associated with the presence of comorbid conditions (aOR = 2.92; 95% CI: 1.09 to 7.80; p = 0.03).

**Conclusion:**

Depression, anxiety, and stress symptoms were present in a significant proportion of home isolated COVID-19 patients in western Nepal and positively associated with the presence of COVID-19 symptoms. In this global COVID-19 pandemic, it is important to provide timely counseling to high-risk groups like those with comorbidities and COVID-19 symptoms to maintain a high level of mental health among home isolated COVID-19 patients.

## 1 Introduction

There is growing evidence that the current COVID-19 pandemic is causing severe mental health effects on people globally [[1], [2], [3], [4]]. Studies conducted globally found a positive association between mental health with the presence of COVID-19 symptoms [5] and comorbid status [6]. The COVID-19 pandemic spread throughout the globe, it instilled significant fear, worry, and concern in the general public, as well as in specific groups such as older individuals, caregivers, and those with underlying health issues [7]. The COVID-19 pandemic is likely to exacerbate social isolation and loneliness which are strongly associated with anxiety, depression, self-harm, and suicide attempts across the lifespan [[8], [9], [10]]. The misinformation from the media, lack of knowledge on COVID-19, lack of effective treatments, and significant economic losses may have resulted in higher levels of depression, anxiety, and stress during the COVID-19 pandemic [11,12]. Anxiety and depression might also be triggered by COVID-19 symptoms such as fever and shortness of breath [13]. Psychological trauma could result in a mixture of emotional surges like nightmares, self-blame, and experiencing recurrent thoughts of trauma [14]. COVID-19 has the potential to cause mental health crises with long-term consequences, particularly in Nepal [15].

The Government of Nepal has reported over 710 thousand COVID-19 cases as of 29th August 2021. About 91.8% of the COVID-19 cases, 36,866 active cases, were isolated at home in Nepal [16]. The impact of the COVID-19 pandemic is huge, and it is already taking a serious toll on the health and economy of the country. In western Nepal, the impact was much higher compared to overall Nepal [17]. The research related to the mental health effects of COVID-19 is limited in Nepal, including the patient's physical well-being. Taking precedence over psychological assessment; this is particularly true in countries like Nepal [18], where infrastructure and psychological screening protocols are severely lacking. To our knowledge, limited studies have determined the depressive symptoms, anxiety symptoms, and stress among home isolated patients infected with COVID-19 in Nepal. Understanding the association between depressive, anxiety, and stress-related symptoms with the presence of COVID-19 symptoms and comorbid status among home isolated COVID-19 patients provides a concrete basis for tailoring and implementing relevant mental health intervention policies [19] to reduce their burden in Nepal.

Therefore, we conducted this study to assess mental health status (depressive, anxiety, and stress-related symptoms) and their association with COVID-19 among COVID-19 patients under home isolation in western Nepal.

## 2 Materials and methods

### 2.1 Study design and setting

We conducted a cross-sectional study among COVID-19 patients under home isolation in the Karnali province of Nepal. Karnali Province is one of the seven provinces of Nepal located in the western part of Nepal. A total of 1,769,788 people are residing in ten districts (Humla, Jumla, Kalikot, Mugu, Surkhet, Dailekh, Salyan, Dolpa, Rukum west and Jajarkot) of Karnali province of Nepal. Karnali Province has a lower human development index (0.469 vs 0.574) compared to overall Nepal [20].

### 2.2 Participants recruitment

The people who tested positive for COVID-19 in the laboratories of Karnali are reported to the Ministry of Social Development (MoSD), Karnali Province. We coordinated with MoSD of Karnali province to identify the COVID-19 positive patients under home isolation. The MoSD provided a list of identified home isolated every day. We recruited 402 eligible home isolated COVID-19 patients in Karnali province from January to May 2021 until the required sample size was met. The sample size was calculated using Cochran's formula [21] assuming a prevalence of depression symptoms at 50% (because it was unknown in this setting), 5.0% absolute error, 5% level of significance, and 5.0% non-response rate. The inclusion criteria applied were: a) PCR positive COVID-19 patients who were isolated at home; b) respondents 18 years or older; c) respondents having a working telephone number and d) respondents who were able to talk on the telephone despite the weakness due to COVID-19. We excluded the respondents who did not answer our call.

### 2.3 Ethical clearance

We obtained ethical approval from the Ethical Review Board (ERB) of Nepal Health Research Council (Ref. No: 1597). We obtained written approval to conduct the study in Karnali province from the Ministry of Social Development (MoSD), Karnali province, Nepal. We also obtained the telephone number of COVID-19 patients from the MoSD, Karnali province and provided detailed information about research to each participant via telephone. We used the telephone number just for research purposes and details of the telephone number were discarded after the completion of the research works. We provide detailed information about this study to all home isolated patients. The participants received ample time to think and ask questions (if needed), and we satisfactorily answered their all queries. We obtained verbal consent from each home isolated COVID-19 patient before enrolling them in this study. Due to COVID-19 travel restrictions and isolation policy with COVID-19 active cases, the ERB approved obtaining verbal consent for this study. We maintained confidentiality and anonymity by keeping all data on password-protected computers.

### 2.4 Data collection

We conducted telephone-based interviews with home isolated COVID-19 patients using pretested structured questionnaires and we entered data directly entered into an online form, created in both Nepali and English languages, in the Kobotoolbox platform [22] during telephone-based interviews (**Supplementary file 1**). In the case of the elderly participants, we interviewed caretakers or family members but for the assessment of depression, anxiety, and stress symptoms we interviewed directly with the home-isolated COVID-19 patients.

### 2.5 Assessment of depression symptoms, anxiety symptoms, and stress

The Nepali version of validated “Depression Anxiety and Stress Scale-21 (DASS-21)” [23] was used to assess depression symptoms, anxiety symptoms, and stress. The internal consistency of the DASS-21 Nepali version was 0.77 for DASS-depression; 0.80 for DASS-anxiety; and 0.82 for DASS-stress, which indicates Cronbach's alpha values. The tool has already been tested in Nepal, its psychometric properties validated, and it was found to be simple, easy to administer, and simple to score. It has been used extensively in previous studies globally as well as in Nepal [24].

The DASS-21 scale contains 21 items (7 items each for depression, anxiety, and stress). Each participant is asked to score every item on a scale from 0 to 3, where 0 indicates “did not apply to me at all” and 3 indicates “apply to me at all”. Total scores for depression, anxiety, and stress are calculated by summing the scores for each scale, multiplied by factor two [23]. We classified patients into mild, moderate, severe, and extremely severe conditions of depression, anxiety, and stress based on the total score within each subdomain as follows [23].

(a) Depression scores were classified as: normal (0–9), mild (10–13), moderate (14–20), severe (21–27), and extremely severe (27+).
(b) Anxiety score was classified as: normal (0–7), mild (8–9), moderate (10–14), severe (15–19), and extremely severe (20+)
(c) Stress score was classified as: normal (0–14), mild (15–18), moderate (19–25), severe (26–33), and extremely severe (34+).

Depression, anxiety, and stress symptoms were further classified as binary (present/absent) outcomes. They were classified as present if at least mild symptoms were present.

### 2.6 Assessment of COVID-19 symptoms and comorbid condition

#### 2.6.1 COVID-19 symptoms

Symptomatic patients were those who reported at least one of the following COVID-19 symptoms in the past one week: fever, cough, sore throat, runny nose, headache, difficulty in breathing, loss of taste, loss of smell, vomiting, diarrhea, muscular pain, abdominal pain, joint pain, chest pain, and irritability/confusion [25].

#### 2.6.2 Comorbid condition

We collected self-reported presence of the following conditions: hypertension, diabetes, heart diseases, thyroid dysfunction, chronic lung disease, chronic renal disease, chronic liver disease, malignancy, pregnancy, post-partum, and immuno-compromised conditions. Those with at least one of the above conditions were categorized as having comorbidity [26].

#### 2.6.3 Assessment of confounding variables

Confounding variables as listed below were selected based on the literature review [27,28].

**Socio-demographic characteristics** included age (in years), sex (male/female), ethnicity (brahmin and chhetri/adhibasi and janajati/tamang, sherpa, rai, limbu, giri and puri), marital status (married/not married), number of formal years of education (in years), family type (joint/ nuclear), monthly family income in Nepalese Rupees (NRs).

**Behavior characteristics** included smoking (never smoker/past smoker/current smoker) and alcohol use in the past month (yes/no) and self-treatment(yes/no). Participants were considered to have self-treatment if they used either traditional methods (like turmeric water, “gurjo” *Tinospora cordifolia, steam water*) or allopathic drugs (like paracetamol) to relieve the symptoms [29].

### 2.7 Statistical analysis

The participants' characteristics were presented in frequency and proportion for categorical variables; mean and standard deviation for normally distributed numerical variables; and median (interquartile range) for non-normally distributed numerical variables. The Clopper-Pearson [30] and Goodman method [31] were used to determine confidence intervals (CI) around binomial and multinomial variables respectively. We utilized a univariate and multivariate logistic regression analysis to determine association of depression, anxiety and stress related symptoms with COVID-19 symptoms. The comorbid conditions were also considered. In the multivariate model, the association of depression, anxiety and stress related symptoms with COVID-19 symptoms and comorbid condition was determined after adjusting for sociodemographic variables (age, gender, ethnicity, marital status, poverty level, education level), smoking status andself-treatment. Crude odds ratio (cOR) and adjusted odds ratio (aOR) with 95% CI were presented. We analyzed data using R-programming software (R-version:4.0.3) [32]**.**

## 3 Results

We approached 521 home isolated COVID-19 patients via telephone, of which 409 (78.5%) responded to our survey and 402 (98.3%) were eligible in the study. Table 1 presents the characteristics of surveyed participants. The age of the participants ranged from 18 to 87 years with a mean age of 36.7 (SD) where about two-thirds were male. The majority were Brahmin and Chhetri (77.4%), followed by Adibasi/Janajati (8.5%). The mean monthly income of the participants was NRs. 44,311.0 ± 34,983.1. The majority of the home isolated patients (91.0%) were non-smokers and 0.7% consumed alcohol in the past one week. About 42.0% experienced COVID-19 related symptoms in the past week and 309 (76.9%) participants were using self-treatment.

**Table 1:** Characteristics of the participants (*n* = 402).

Characteristics (n = 402)	n (%)
Sex	
Male	271 (67.4)
Female	131 (32.6)
Age (in years), Mean ± SD	36.7 ± 12.8 min: 18, max: 87
Education (in years)	12.2 ± 3.7 min: 0, max: 19
Ethnicity	
Brahmin and Chhetri	311 (77.4)
Adhibasi/Janjati	34 (8.5)
Other (Tamang, Rai, Gurung)	57 (14.2)
Marital status	
Married	296 (74.8)
Not married	106 (26.4)
Type of Family	
Nuclear	335 (83.3)
Joint	67 (16.7)
Monthly family income (NRs), Mean ± SD	44,311.0 ± 34,983.1 min: 2000.0 max: 300000.0
Presence of Comorbidities	42 (10.4)
Presence of COVID-19 symptoms	167 (41.5)
Self-treatment	309 (76.9)
Smoking	
Never smoker	366 (91.0)
Current smoker	20 (5.0)
Past smoker	16 (4.0)
Alcohol use within past one month	3 (0.7)

**n:** frequency, **min:** minimum; **max:** maximum, **SD:** standard deviation; **NRs:** Nepalese Rupees.

**Comorbidities:** hypertension, diabetes, heart diseases, thyroid dysfunction, chronic lung disease, chronic renal disease, chronic liver disease, malignancy, pregnancy, post-partum, and immuno-compromised conditions.

**COVID-19 symptoms:** fever, cough, sore throat, runny nose, headache, difficulty in breathing, loss of taste, loss of smell, vomiting, diarrhoea, muscular pain, abdominal pain, joint pain, chest pain, and irritability/confusion.

**Fig. 1:**
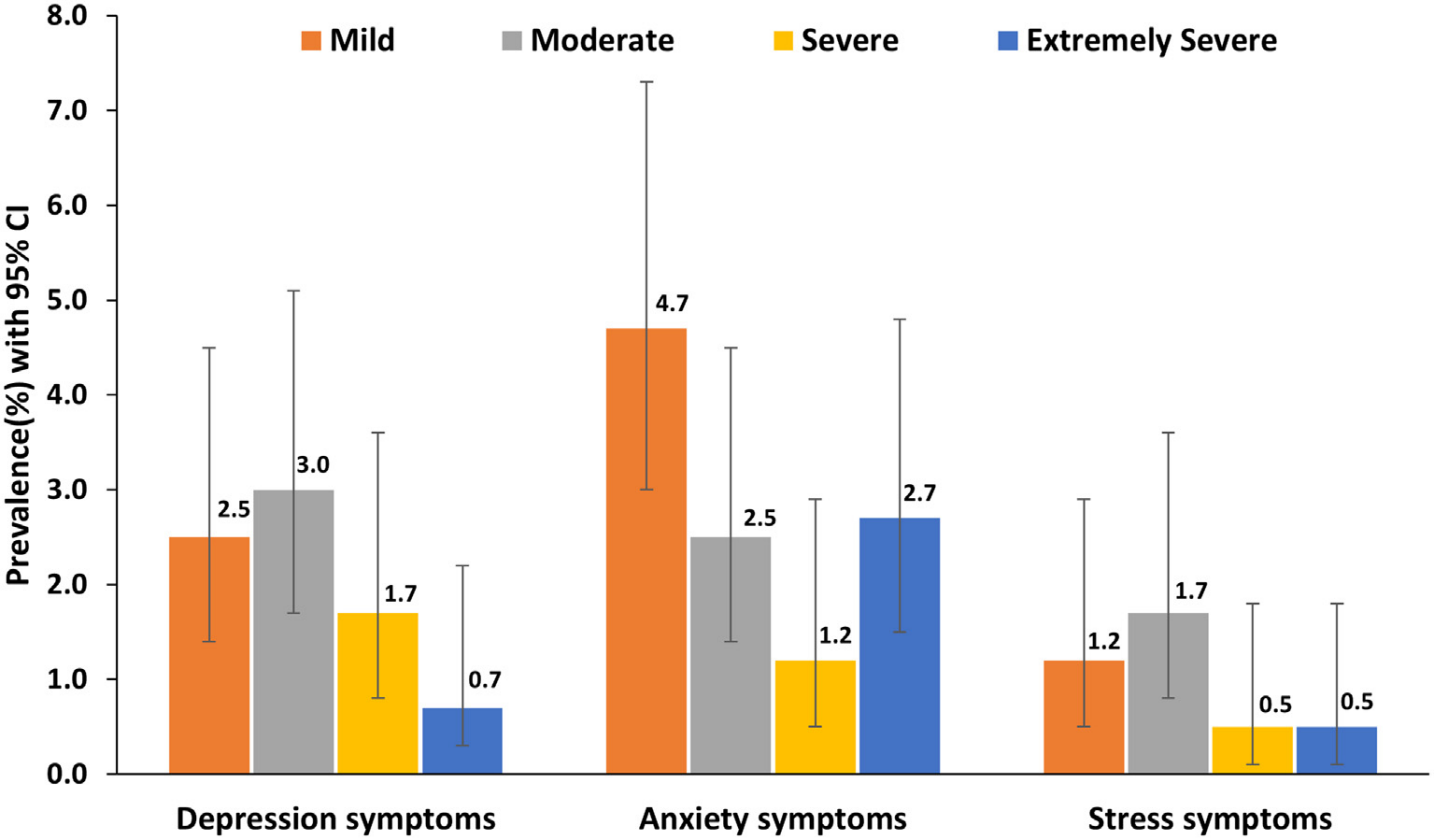
Prevalence of depression, anxiety, and stress symptoms (*n* = 402).

Fig. 1 shows the prevalence of different levels of the severity of depression, anxiety, and stress symptoms among home isolated COVID-19 patients of Karnali province. Of the total participants, 8.0% (95% CI: 5.5 to 11.1) had depression symptoms, 11.2% (95% CI: 8.3 to 14.7) had anxiety symptoms and 4.0% (95% CI: 2.3 to 6.4) had stress symptoms.

Table 2 shows the univariate and multivariate logistic regression to determine association of presence of comorbidity and COVID-19 symptoms with depression symptoms among home isolated COVID-19 patients in Karnali province. Univariate analysis showed significant positive association of depression symptoms with both presence of comorbid condition (cOR = 8.05 (95% CI: 3.61 to 17.92); *p* < 0.01) and COVID-19 symptoms (cOR = 4.01 (95% CI: 1.80 to 8.91); *p* < 0.01). Symptomatic patients had twice higher odds of having depression symptoms compared to those who were asymptomatic (aOR: 2.86; 95% CI: 1.10 to 7.44, *p*-value:0.03) after adjusting age, gender, monthly family income, marital status, type of family, ethnicity, education level, presence of the comorbid condition, smoking status, and presence of self-treatment.

**Table 2:** Factors associated with depression symptoms among home isolated COVID-19 patients Karnali province (*n* = 402).

Variables	Depression symptoms n(%)	cOR (95% CI)	p-value	aOR (95%CI)⁎	*p*-value
Presence of Comorbidities					
Absent	19(5.3)	1		1	
Present	13(31.0)	**8.05 (3.61–17.92)**	**<0.01**	2.96 (0.99–8.85)	0.05
Presence of COVID-19 symptoms					
No	9(3.8)	1		1	
Yes	23(13.8)	**4.01((1.80–8.91)**	**<0.01**	**2.86 (1.10–7.44)**	**0.03**

**n:** frequency; **cOR:** crude Odds Ratio; **aOR:** adjusted Odds Ratio; **CI:** Confidence interval.

**Bold indicates statistically significant at 95%CI.**

⁎ **Adjusting variables:** age, gender, monthly income, marital status, family type, ethnicity, education level, smoking status, and presence of self-treatment.

Table 3 shows the univariate and multivariate logistic regression to determine association of presence of comorbidity and symptoms with anxiety symptoms among home isolated COVID-19 patients in Karnali province. In univariate analysis and multivariate analysis, anxiety symptoms were positively associated with presence of comorbidity and COVID-19 symptoms. The participants with at least one COVID-19 symptoms in the past week were four times more likely (aOR: 3.81; 95% CI: 1.62–8.93; *p*-value:<0.01) to have anxiety symptoms compared to those without symptoms and participants having comorbid conditions were three times more likely (aOR: 2.92; 95% CI: 1.09–7.80; *p*-value: 0.03) to have anxiety symptoms after adjusting for age, gender, monthly family income, marital status, ethnicity, education level, smoking status and presence of self-treatment.

**Table 3:** Factors associated with anxiety symptoms among home isolated COVID-19 patients Karnali province (*n* = 402).

Variables	Anxiety symptoms n (%)	cOR (95% CI)	p-value	aOR(95%CI) ⁎	p-value
Comorbidities					
Absent	27(7.5)	1		1	
Present	18(42.9)	**9.25(4.47–19.12)**	**<0.01**	**2.92(1.09–7.80)**	**0.03**
COVID-19 symptoms					
No	13(5.5)	1		1	
Yes	32(19.2)	**4.05(2.05–7.98)**	**<0.01**	**3.81(1.62–8.93)**	**<0.01**

**n:** frequency; **cOR:** crude Odds Ratio; **aOR:** adjusted Odds Ratio; **CI:** Confidence interval.

**Bold indicates statistically significant at 95% CI.**

⁎ **Adjusting variables:** age, gender, monthly family income, marital status, type of family, ethnicity, education level, smoking status and self-treatment.

Table 4 shows the univariate and multivariate logistic regression to determine association of presence of comorbidity and symptoms with stress symptoms among home isolated COVID-19 patients in Karnali province. In univariate analysis, stress symptoms was positively associated with presence of comorbidity and symptoms. The participants with at least one COVID-19 symptoms in the past week were 7.78 times more likely (aOR: 7.78; 95% CI: 1.43–42.28; *p*-value:<0.02) to have stress symptoms compared to those without any COVID-19 symptoms after adjusting for age, gender, monthly family income, marital status, ethnicity, education level, smoking status, and presence of self_treatment.

**Table 4:** Factors associated with stress among home isolated COVID-19 patients in Karnali province (*n* = 402).

Variables	Stress symptoms n(%)	cOR (95% CI)	p-value	aOR(95%CI) ⁎	p-value
Comorbidities					
Absent	10(2.8)	1		1	
Present	6(14.3)	**5.83(2.00–16.98)**	**0.01**	1.22(0.24–6.26)	0.81
COVID-19 symptoms					
No	2(5.5)	1		1	
Yes	14(19.2)	**10.66(2.39–47.56)**	**<0.01**	**7.78 (1.43–42.28)**	**0.02**

**n:** frequency; **cOR:** crude Odds Ratio; **aOR:** adjusted Odds Ratio; **CI:** Confidence interval.

**Bold indicates statistically significant at 95%CI.**

⁎ **Adjusting variables:** age, gender, monthly family income, marital status, type of family, ethnicity, education level, smoking status, and self-treatment.

## 4 Discussion

Our results showed that depression, anxiety, and stress related symptoms were prevalent among the home isolated patients of Karnali province. The most common was anxiety symptoms affecting 11.0% of the study population, followed by depression affecting 8.0% and stress affecting 4% of the study population. Depression, anxiety, and stress were positively associated with the presence of COVID-19 symptoms. In addition, anxiety related symptoms were positively associated with the presence of comorbid conditions.

The prevalence of depression and anxiety symptoms were higher in home isolated COVID-19 patients in Nepal, compared to the general population. The prevalence of depression and anxiety is estimated to be 3.2% and 3.6%, respectively [33]. A study conducted among fever clinic attendants in Nepal reported a similar prevalence of depression anxiety and stress - 7.0%, 14.0%, and 5.0%, respectively [15]. This higher prevalence might be attributable to experience due to COVID-19 infection [34]. The SARS-CoV-2 (COVID-19) pandemic has resulted in increased levels of anxiety, depression, and stress around the globe because of different factors like lockdown, grief, survivor guilt, unemployment, insecure employment and economic loss [34].

The prevalence of depression symptoms, anxiety symptoms, and stress were lower in our study compared to the COVID-19 patients in other countries. A study conducted among hospitalized and home isolated COVID-19 patients in Sharkia Governorate, Egypt reported the prevalence of depression and anxiety reported to be 69.7% and 32.6% respectively among home isolated COVID-19 patients [35]. Similarly, a study conducted in Iran reported the prevalence of “extremely severe” depression, anxiety and stress symptoms to be 54.3% and 97.3%, 46.6% among positive patients [36]. According to a meta-analysis, the pooled prevalence of depression and anxiety symptoms among COVID-19 patients were 45% (95% CI: 37–54%), and 47% (95% CI: 37–57%) respectively [37] which was higher compared to the findings of our study among home isolated COVID-19 patients [37]. This vast variance in prevalence might be explained by differences in study participants, circumstances, or the use of different definitions and tools in the studies.

In our study, the presence of COVID-19 symptoms was associated with the increased odds of depression symptoms, anxiety symptoms, and stress. A cohort study in a Brazilian city showed a positive association of COVID-19 symptoms with depression, anxiety, and post-traumatic stress [38]. A study showed that anxiety and depression might be triggered by COVID-19 related symptoms such as fever and shortness of breath [13]. In our study, anxiety symptoms and stress were found to be associated with having comorbidity, which may be because of awareness among the participants that comorbid patients have a higher risk of mortality due to COVID-19 [39,40] which contributed to their fear of morbidity and mortality thus resulting in stress, anxiety symptoms, and depression symptoms [34].

Our study has several strengths. This is one of the first studies reporting the prevalence and factors associated with depression symptoms, anxiety symptoms, and stress among home isolated COVID-19 patients in Nepal. We used a pre-tested validated standard Nepali-translated DASS-21 scale to estimate depression symptoms, anxiety symptoms and stress levels among participants of the study. The collected data were cross-checked for cleanliness and errors at the end of every week. However, there were some limitations. First, we could not establish the directionality of the risk associations because of the cross-sectional nature of the study. Second, generalizability of the study is limited to western Nepal rather than overall Nepal. Third, comorbidity status of the patients was self-reported which may have been underestimated since some may be undiagnosed comorbid conditions. Self-reported symptomatic status of home isolated patients may have been underestimated because of risk of recall bias. Fourth, pre-existing mental health status like depression, anxiety, and stress was not assessed. Finally, we could not measure and adjust childhood adversity, social support, perceived stress, perceived stigma and occupation-related factors like job insecurity and family work balance that can potentially affect mental health resulting in depression symptoms, anxiety symptoms, and stress [41].

## 5 Conclusion

The prevalence of depression symptoms, anxiety symptoms, and stress among home isolated COVID-19 patients of Karnali province were common. They were positively associated with the presence of COVID-19 symptoms whereas anxiety symptoms with comorbid status. One key implication of this study is that, in this global COVID-19 pandemic, Nepal government should provide timely counseling and psychological support to those who are already affected with depression symptoms, anxiety symptoms and stress; and those under high-risk like those with comorbidities and COVID-19 symptoms to maintain a high level of mental health among home isolated COVID-19 patients.

## Funding

This study was conducted from a project entitled “COVID-19: Strengthening Provincial Level COVID Response in Nepal” which was funded by the Bill Gates and Melinda Gates foundation (INV-021518).

## Declaration of Competing Interest

We/ Authors declare no conflict of interests.

## Data Availability

All data are fully available without restriction.

## Appendix A Supplementary data

Supplementary material Image 1

## References

[1] Torales J., O’Higgins M., Castaldelli-Maia J.M., Ventriglio A. (2020). The outbreak of COVID-19 coronavirus and its impact on global mental health. Int J Soc Psychiatry.

[2] Zandifar A., Badrfam R., Yazdani S., Arzaghi S.M., Rahimi F., Ghasemi S. (2020). Prevalence and severity of depression, anxiety, stress and perceived stress in hospitalized patients with COVID-19. J Diabetes Metab Disord.

[3] Huang Y., Zhao N. (2020). Generalized anxiety disorder, depressive symptoms and sleep quality during COVID-19 outbreak in China: a web-based cross-sectional survey. Psychiatry Res.

[4] Moghanibashi-Mansourieh A. (2020). Assessing the anxiety level of Iranian general population during COVID-19 outbreak. Asian J Psychiatr.

[5] Perlis R.H., Ognyanova K., Santillana M., Baum M.A., Lazer D., Druckman J. (2021). Association of Acute Symptoms of COVID-19 and Symptoms of Depression in Adults. JAMA Netw Open.

[6] Sayeed A., Kundu S., Al Banna M.H., Christopher E., Hasan M.T., Begum M.R. (2020). Mental health outcomes of adults with comorbidity and chronic diseases during the COVID-19 pandemic: a matched case-control study. Psychiatr Danub.

[7] Mental health and COVID-19 (2021). https://www.euro.who.int/en/health-topics/health-emergencies/coronavirus-covid-19/publications-and-technical-guidance/mental-health-and-covid-19.

[8] Holmes E.A., O’Connor R.C., Hugh Perry V., Tracey I., Wessely S., Arseneault L. (2020). Multidisciplinary research priorities for the COVID-19 pandemic: a call for action for mental health science. Lancet Psychiatry.

[9] Elovainio M., Hakulinen C., Pulkki-Råback L., Virtanen M., Josefsson K., Jokela M. (2017). Contribution of risk factors to excess mortality in isolated and lonely individuals: an analysis of data from the UK Biobank cohort study. Lancet Public Health.

[10] Matthews T., Danese A., Caspi A., Fisher H.L., Goldman-Mellor S., Kepa A. (2019). Lonely young adults in modern Britain: findings from an epidemiological cohort study. Psychol Med.

[11] Mautong H., Gallardo-Rumbea J.A., Alvarado-Villa G.E., Fernández-Cadena J.C., Andrade-Molina D., Orellana-Román C.E. (2021). Assessment of depression, anxiety and stress levels in the Ecuadorian general population during social isolation due to the COVID-19 outbreak: a cross-sectional study. BMC Psychiatry.

[12] Zarocostas J. (2020). How to fight an infodemic. Lancet..

[13] Fitzgerald P.J. (2020). Serious infection may systemically increase noradrenergic signaling and produce psychological effects. Med Hypotheses.

[14] Nesterko Y., Jäckle D., Friedrich M., Holzapfel L., Glaesmer H. (2020). Factors predicting symptoms of somatization, depression, anxiety, post-traumatic stress disorder, self-rated mental and physical health among recently arrived refugees in Germany. Confl Health.

[15] Devkota H.R., Sijali T.R., Bogati R., Ahmad M., Shakya K.L., Adhikary P. (2020). The Impact of COVID-19 on Mental Health outcomes among hospital fever clinic attendants across Nepal: A community-based cross-sectional study. medrxiv..

[16] Coronavirus disease (COVID-19) outbreak updates & resource materials – Health Emergency Operation Center. [cited 7 Jun 2021]. Available:https://heoc.mohp.gov.np/update-on-novel-corona-virus-covid-19/

[17] replication-receiver (12 Mar 2020). UNDP [Internet].

[18] Luitel N.P., Jordans M.J., Adhikari A., Upadhaya N., Hanlon C., Lund C. (2015). Mental health care in Nepal: current situation and challenges for development of a district mental health care plan. Confl Health.

[19] Steenblock C., Schwarz P.E.H., Perakakis N., Brajshori N., Beqiri P., Bornstein S.R. (2022). The interface of COVID-19, diabetes, and depression. Discov Ment Health.

[20] Paudel T., Amgain K., Sanjel S. (2018). Health scenario of Karnali Province. J Karnali Acad Health Sci.

[21] Cochran W.G., Cochran W.G. (1977). https://books.google.com/books/about/Sampling_Techniques.html?hl=&id=8Y4QAQAAIAAJ.

[22] KoboToolbox. In: KoboToolbox [Internet]. [cited 2 Dec 2021]. Available:https://www.kobotoolbox.org/

[23] Lovibond S.H., Lovibond P.F. (2011).

[24] Tonsing K.N. (2014). Psychometric properties and validation of Nepali version of the Depression Anxiety Stress Scales (DASS-21). Asian J Psychiatr.

[25] CDC (26 Oct 2022). Symptoms of COVID-19. In: Centers for disease control and prevention [Internet]. https://www.cdc.gov/coronavirus/2019-ncov/symptoms-testing/symptoms.html.

[26] CDC (1 Dec 2022). People with certain medical conditions. In: Centers for disease control and prevention [Internet]. https://www.cdc.gov/coronavirus/2019-ncov/need-extra-precautions/people-with-medical-conditions.html.

[27] Souza A.S.R., Souza G.F.A., Souza G.A., Cordeiro A.L.N., Praciano G.A.F., ACS De Alves (2021). Factors associated with stress, anxiety, and depression during social distancing in Brazil. Rev Saude Publica.

[28] Risal A., Manandhar K., Linde M., Steiner T.J., Holen A. (2016). Anxiety and depression in Nepal: prevalence, comorbidity and associations. BMC Psychiatry.

[29] Chi S., She G., Han D., Wang W., Liu Z., Liu B. (2016). Genus Tinospora: Ethnopharmacology, Phytochemistry, and Pharmacology. Evid Based Complement Alternat Med.

[30] Clopper C.J., Pearson E.S. (1934). The use of confidence or fiducial limits illustrated in the case of the binomial. Biometrika..

[31] Goodman L.A. (1965). On Simultaneous Confidence Intervals for Multinomial Proportions. Technometrics..

[32] R Core Team (2020). R: A language and environment for statistical computing. R Foundation for Statistical Computing. Vienna, Austria. https://www.R-project.org/.

[33] World Health Organization (2017). https://apps.who.int/iris/bitstream/handle/10665/254610/WHO-MSD-MER-2017.2-eng.pdf.

[34] Brenner M.H., Bhugra D. (2020). Acceleration of anxiety, depression, and suicide: secondary effects of economic disruption related to COVID-19. Front Psych.

[35] Mohamed A.E., Yousef A.M. (2021). Depressive, anxiety, and post-traumatic stress symptoms affecting hospitalized and home isolated COVID-19 patients: a comparative cross-sectional study. Middle East Curr Psychiatry.

[36] Moayed M.S., Vahedian-Azimi A., Mirmomeni G., Rahimi-Bashar F., Goharimoghadam K., Pourhoseingholi M.A. (2021). Depression, anxiety, and stress among patients with COVID-19: A cross-sectional study. Adv Exp Med Biol.

[37] Deng J., Zhou F., Hou W., Silver Z., Wong C.Y., Chang O. (2021). The prevalence of depression, anxiety, and sleep disturbances in COVID-19 patients: a meta-analysis. Ann N Y Acad Sci.

[38] Ismael F., Bizario J.C.S., Battagin T., Zaramella B., Leal F.E., Torales J. (2021). Post-infection depressive, anxiety and post-traumatic stress symptoms: A prospective cohort study in patients with mild COVID-19. Prog Neuropsychopharmacol Biol Psychiatry.

[39] Islam M.Z., Riaz B.K., Islam A.N.M.S., Khanam F., Akhter J., Choudhury R. (2020). Risk factors associated with morbidity and mortality outcomes of COVID-19 patients on the 28th day of the disease course: a retrospective cohort study in Bangladesh. Epidemiol Infect.

[40] Noor F.M., Islam M.M. (2020). Prevalence and associated risk factors of mortality among COVID-19 patients: a meta-analysis. J Community Health.

[41] Sheikh M.A. (2017). Confounding and statistical significance of indirect effects: childhood adversity, education, smoking, and anxious and depressive symptomatology. Front Psychol.

