# Supplementary material for: Prevalence and factors associated with depression, anxiety, and stress symptoms among home isolated COVID-19 patients in Western Nepal
Source: Dialogues Health. 2022 Dec 5;2:100090. doi: 10.1016/j.dialog.2022.100090 (PMC9721187; doi:10.1016/j.dialog.2022.100090)
Supplement: Supplementary file 1 — Supplementary material [file mmc1.pdf]

Final Situational Analysis of Home Isolation for Data collection

अन्तर्वार्ता लिने व्यक्तिको पुरा नाम

Consent

म तपाईंलाई सामाजिक बिकास मन्त्रालयको तर्फ बाट फोन गर्दैछु । आज तपाईंलाई फोन गर्नु को उद्देश्य होम आइसोलेसन को व्यवस्थापन कस्तो छ भन्ने कुराको जानकारी लिन हो । तपाईं सँग म २० मिनेट जती कुरा गर्ने छु । यस कुराकानीबाट आएका जानकारीहरु सम्बन्धित स्वास्थ्य तथा प्रशासनिक निकायलाई दिन्छौँ ।म तपाईले उपलब्ध गराउने जानकारीको सुरक्षा गर्दछु र तपाईको नाम कुनै पनि रिर्पोट वा अन्य प्रकाशन र प्रस्तुतीकारणमा उल्लेख गर्ने छैन । तपाईंलाई यदी कुरा गर्न अप्ठ्यारो छ भने, नगर्नु भए पनि हुन्छ।

यो कुराकानी गर्नको लागी तपाईं सहमत हुनु हुन्छ ?

- सहमत छु
- सहमत छैन

किन सहमत हुनु हुँदैन ? कारण भनिदिनुस

Survey

» General Information

नाम

मोबाइल नम्बर

जिल्ला

पालिका

घरको नम्बर

Household number

वडा न

Swab collection गरेको मिती

Date of PCR

yyyy-mm-dd

नतिजा थाहा भएको मिती

yyyy-mm-dd

घर मुली को नाम

who has number

घर मुलीको नम्बर

» A. Socio-demographic Characteristics

तपाइको उमेर कति भयो?

(years)

उत्तरदाताको लिङ्ग

- पुरूष
- महिला
- अन्य

जम्मा तपाइले कति बर्ष पूर्ण समय अध्ययन गर्नु भएको छ ?

numeric from 1-12 number of classes; Bachelors 3 years = 12+3; Bachelors 4 years = 12+4; Masters = 12+3 or 4 + 2

वैवाहिक स्थिति

- ◯ विवाहित
- ◯ अविवाहित
- ◯ विधवा
- ◯ सम्बन्धविच्छेद

जातीयता

- ◯ ब्राह्मण/क्षेत्री
- ◯ तराई/ मधेसी अन्य जातीय
- ◯ नेवार
- ◯ जनजाती/आधिवासी
- ◯ मुस्लिम
- ◯ दलित
- ◯ अन्य

अन्य (उल्लेख गर्नुहोस्)

तपाइको मुख्य पेशा के हो ?

- ◯ जागिर (सरकारी)
- ◯ जागिर (गैरसरकारी)
- ◯ व्यापार व्यवसाय
- ◯ घरायसी
- ◯ विधार्थी
- ◯ ज्यालादारी / मजदुरी
- ◯ अवकाश
- ◯ बेरोजगार (काम गर्न सक्ने)
- ◯ बेरोजगार (काम गर्न नसक्ने
- ◯ स्वास्थ्य कर्मी
- ◯ पशु चिकित्सक
- ◯ स्वास्थ्य प्रयोगशाला कर्मचारी
- ◯ अन्य (उल्लेख गर्नुहोस्)

यदी अन्य, (Specify)

तपाइको परिवारको मासिक आम्दानी कति होला?

____

तपाइको परिवारको संरचना कस्तो खालको छ?

- ◯ एकल
- ◯ संयुक्त

तपाइको परिवारमा जम्मा कति जना सदस्यहरु हुनुहुन्छ

____

हाल तपाईं सहित तपाइको परिवारका कति जना सदस्य कोभिड-१९ बाट संक्रमित हुनुहुन्छ?

____

तपाईको परिवारमा पहिले कति जना सदस्य कोभिड-१९ बाट संक्रमित हुनुभएको थियो?

____

तपाई पोजिटिभ भए पछि कति जना परिवारका सदस्यहरुको परिक्षण भयो ?

____

कसैले कन्ट्याक्ट ट्रेसिङ्गो लागि फोन गरेको छ ?

- ◯ छ
- ◯ छैन

हाल तपाई बस्नु भएको घरको स्वामित्तो कस्को हो ?

- ◯ आफ्नो
- ◯ भाडाको

» B. Physical Health Status

हाल लक्षण देखिएको

- ◯ छ
- ◯ छैन

यदी छैन भने, गएको ४ हप्तामाकोभिड १९ को कुनै लक्षणहरू देखिएको छ?

- ◯ छ
- ◯ छैन

यदी छ भने के-के छ (multiple response)

- [ ] ज्वरो
- [ ] कमजोरी
- [ ] खोकी
- [ ] घाटी दुख्ने
- [ ] पातलो सिँगान बग्ने
- [ ] स्वास फेर्न गार्हो
- [ ] पखाला
- [ ] वाकवाक लाग्नु
- [ ] टाउकोदुख्ने
- [ ] झर्को/अन्यौल
- [ ] स्वाद हराउनु
- [ ] बास्ना हराउने
- [ ] मांशपेशी दुखाइ
- [ ] छाती दुखाइ
- [ ] पेट दुखाइ
- [ ] जोर्नी दुखाइ
- [ ] अन्य (उल्लेख गर्नुहोस्)

यदी अन्य, (Specify)

___

हजुरलाइ पहिलो लक्षण के देखिएको थियो ? (single response)..

- [ ] ज्वरो
- [ ] कमजोरी
- [ ] खोकी
- [ ] घाटी दुख्ने
- [ ] पातलो सिँगान बग्ने
- [ ] स्वास फेर्न गार्हो
- [ ] पखाला
- [ ] वाकवाक लाग्नु
- [ ] टाउकोदुख्ने
- [ ] झर्को/अन्यौल
- [ ] स्वाद हराउनु
- [ ] बास्ना हराउने
- [ ] मांशपेशी दुखाइ
- [ ] छाती दुखाइ
- [ ] पेट दुखाइ
- [ ] जोर्नी दुखाइ
- [ ] अन्य (उल्लेख गर्नुहोस्)

यदी अन्य, (Specify)

____

पहिलो लक्षणको सुरुवातको मिति..

yyyy-mm-dd

तपाईका लक्षणहरु कस्तो हुँदैछन्?

- ( ) उस्तै छ
- ( ) राम्रो
- ( ) नराम्रो
- ( ) थाहा छैन

तपाइलाइ स्वास्थ्यसँग सम्बन्धित कुनै अवस्था, समस्या वा रोगहरू केहि छन ?

- [ ] गर्भावस्था (त्रैमासिक)
- [ ] सुत्केरीवस्था(<६हप्ता)
- [ ] मुटु सम्बन्धी रोग, रक्तचाप सहित
- [ ] मधुमेह
- [ ] कलेजोको रोग
- [ ] नसा सम्बन्धी रोग
- [ ] मृगौलाको रोगहरु
- [ ] कुपोषण
- [ ] अटोइम्युनरोगहरु
- [ ] इम्युनोडेफिशियन्सी, एच.आईभी सहित
- [ ] म्यालिगनेन्शी
- [ ] फोक्सोको दीर्घरोग/दम/धमनी
- [ ] कुनैपनि छैन
- [ ] अन्य

त्रैमासिक (Trimester)

____

अन्य (उल्लेखगर्नु)

____

के तपाई कोरोनाको लागि कुनै उपचार लिदै हुनुहुन्छ?

- ( ) छ
- ( ) छैन

यदी छ भने, कस्तो खाल्को उपचार ?

- ( ) घरेलु उपचार
- ( ) औषधी उपचार
- ( ) दुबै

कस्तो प्रकारको घरेलु उपचार

- [ ] बाफ लिने
- [ ] बेसार पानी खाने
- [ ] गुर्जो खाने
- [ ] अन्य

अन्य (उल्लेखगर्नु)

कस्तो प्रकारको औषधि

- [ ] Antipyretic
- [ ] Analgesics
- [ ] Anti-histaminic
- [ ] Cough syrup
- [ ] ORS
- [ ] Antibiotic
- [ ] Supplements
- [ ] Others

Others (Specify)

» C. Home Isolation Part 1

घरको प्रकार

- ○ कच्चा
- ○ पक्का
- ○ कच्चा - पक्का

के तपाइको घरमा छुटैबस्नको लागि पर्याप्त कोठाको व्यवस्था छ?

- ○ छ
- ○ छैन
- ○ थाहा छैन

कति कोठाहरु छन् ?

Except Kitchen

के तपाई एक्ले छुट्टै कोठामा बस्दै हुनुहुन्छ ?

- ○ छ
- ○ छैन
- ○ थाहा छैन

तपाईंको कोठामा कतिवटा झ्यालहरू छन्?

____

यदि एक भन्दा बढी झ्यालहरु छन भने, ति झ्यालहरु विपरित स्थानमा छन ? (Cross ventilation)

- ◯ छ
- ◯ छैन
- ◯ थाहा छैन

के तपाईको झ्यालबाट सीधा कोठामा सूर्यको किरण आउछ?

- ◯ छ
- ◯ छैन
- ◯ थाहा छैन

के तपाई छुट्टै toilet/बाथरूमको प्रयोग गर्नुहुन्छ ?

- ◯ छ
- ◯ छैन
- ◯ थाहा छैन

के तपाईसँग बाहिर बाट अरु कसैले खाना तथा खाद्यसामग्री घरमै ल्याइदिने सुविधा छ ?

- ◯ छ
- ◯ छैन
- ◯ थाहा छैन

यदी छ भने कस्ले ?

- ◯ स्वास्थ्य कार्यालय
- ◯ पालिका
- ◯ परिवारका सदस्य
- ◯ छिमेकी/साथी
- ◯ आफन्त
- ◯ अन्य

यदी अन्य, (Specify)..

____

के तपाईसँग आवश्यक परेको बेला बाहिरबाट अरु कसैले औषधी ल्याइदिने सुविधा छ?

- छ
- छैन
- थाहा छैन

यदी छ भने कस्ले ?

- स्वास्थ्य कार्यालय
- पालिका
- परिवारका सदस्य
- छिमेकी/साथी
- आफन्त
- अन्य

यदी अन्य, (Specify)

आकस्मिक उपचारको आवश्यक पर्दा के तपाई सँग यातायात को पहुँच छ ?

- छ
- छैन
- थाहा छैन

तपाइको हेरचाह गर्न घरमा को उपलब्ध हुनुहुन्छ?

- परिवारका सदस्यहरु
- कोही छैन
- नोकर
- छिमेकी
- अन्य

यदी अन्य, (Specify)

तपाईले कोठाको फोहोर व्यवस्थापन कसरी गर्नुहुन्छ?

- आफैले व्यवस्थापन गर्छु
- अरुले फोहोर लिन आउछ

आफैले कसरी व्यवस्थापन गर्नु हुन्छ ?

- [ ] जलाउने
- [ ] जमीनमा पुर्ने
- [ ] जथाभावी फाल्ने
- [ ] अन्य

अन्य (उल्लेखगर्नु)

____

के तपाईसँग संक्रमण रोकथामकोलागि निम्न आधारभूत स्वस्थ्य सामाग्रीहरु उपलब्ध छन्:	छ	छैन
face guards	○	○
Oxymeter	○	○
Sanitizer	○	○
Thermometer	○	○
Disinfectant	○	○

» D. Home Isolation Part 2

Home Isolation Part 2	सधैँ	धेरै जस्तो	कहिले काहि	कहिले पनि गर्दिन
1. के तपाइँ खोक्ने वा छींकने बेलामा नाकलाई टिश्युले वा कुहिनोले ढाकनुहुन्छ ?	○	○	○	○
2. तपाईले प्रयोग गर्ने गिलास, कप, थाल धोएर अरु कसैले पनि प्रयोग गर्नुहुन्छ ?	○	○	○	○
3. तपाईं घर भित्र अरु व्यक्तिको नजिक बस्दा मास्क प्रयोग गर्नुहुन्छ?	○	○	○	○
4. के तपाईंले श्वासप्रश्वासको स्राव (राल, खकार, सिगान) सँगको सम्पर्क पछि आफ्नो हातहरू धुनुहुन्छ?	○	○	○	○
5. के तपाईं परिवारका सदस्यहरूसँग २ मिटर दूरी कायम गर्नुहुन्छ?	○	○	○	○
6. के तपाइँ डिसइन्फेक्टेन प्रयोग गरेर कोठा सफा गर्नुहुन्छ ?	○	○	○	○
7. के तपाइँ thermometer ले ज्वोरो नाप्नु हुन्छ ?	○	○	○	○
8. के तपाइँ घरबाट बाहिर निस्किनु हुन्छ ?	○	○	○	○
9. के तपाइँ स्वास्थ्य कर्मीसंग नियमित रुपमा सम्पर्क राख्नु हुन्छ ?	○	○	○	○

एक दिनमा कति पटक हात धुनुहुन्छ?

____

» E. Short Form (SF-8)

1. गएको हप्तामा, तपाईले आफ्नो स्वस्थालाई कसरी मापन गर्नुहुन्छ ?

- अति नै राम्रो
- धेरै राम्रो
- राम्रो
- ठिकै
- नराम्रो
- धेरै नराम्रो

2. गएको हप्तामा, तपाईको स्वास्थ्य स्थितिले तपाईको शारीरिक क्रियाकलाप जस्तै कि हिडडुल गर्ने, भर्याङ्ग चड्ने मा कतिको असर पारेको थियो?

- केहि असर परेको थिएन
- अलि अलि असर परेको थियो
- ठिक्कै असर परेको थियो
- धेरै नै असर परेको थियो
- म केहि पनि शारीरिक काम गर्न सक्दिन

3.गएको हप्तामा, तपाईको शारीरिक स्वास्थ्य अवस्थाको कारण तपाईको दैनिक क्रियाकलापमा कतिको असर परेको थियो ?

- केहि असर परेको थिएन
- अलि अलि असर परेको थियो
- ठिक्कै असर परेको थियो
- धेरै नै असर परेको थियो
- दैनिक काम नै गर्न सक्दिन

4. गएको हप्तामा, तपाईले कतिको शारीरिक पिडा जस्तै टाउको दुख्ने, जीउ दुख्ने महसुश गर्नु भयो ?

- पटक्कै छैन
- अति नै कम
- कम
- ठिक्कै
- धेरै
- अति नै धेरै

5. गएको हप्तामा, तपाई कतिको फुर्तिलो हुनुहुन्थ्यो?

- एकदम धेरै
- धेरै नै
- अलि अलि
- एकदमै कम
- कति पनि थिएन

6. गएको हप्तामा, तपाईको शारीरिक स्वास्थ्य स्थिति वा भावनात्मक समस्याको कारणले तपाईको परिवार वा साथीभाई संग भेटघाट, मेलमिलाप, भोज-भतेर आदीमा कतिको असर परेको छ ?

- केहि असर परेको थिएन
- अलि अलि असर परेको थियो
- ठिक्कै असर परेको थियो
- धेरै नै असर परेको थियो
- दैनिक काम नै गर्न सक्दिन

7. गएको हप्तामा, तपाईको भावनात्मक समस्या जस्तै कि के गरौ के गरौ हुने, चिन्ता, उदास हुने वा झर्को लाग्ने समस्याले कतिको सताएको छ?

- केहि सताएको छैन
- अलि अलि सताएको छ
- ठिक ठिकै सताएको छ
- धेरै नै सताएको
- एकदमै धेरै सताएको छ

8.गएको हप्तामा, तपाईको ब्यतिगत तथा भावनात्मक समस्याले गर्दा तपाईले आफ्नो काम, पढाइ वा अरु दैनिक क्रियाकलाप कतिको छुटाउनु भयो?

- केहि असर परेको थिएन
- अलि अलि असर परेको थियो
- ठिक्कै असर परेको थियो
- धेरै नै असर परेको थियो
- दैनिक काम नै गर्न सक्दिन

» F. Mental Status (DASS-21):

**Please read each statement and circle a number 0, 1, 2 or 3 which indicates how much the statement applied to you over the past week. There are no right or wrong answers. Do not spend too much time on any statement. कृपया प्रत्येक कथन/वक्तव्य पढ्नुहोस् र बितेको हप्तामा उक्त बयान/भनाइ तपाईंलाई कति मिल्छ वा लागु भएकोछ भनेर संकेत संख्या ०, १, २ वा ३ मा circle गर्नुहोस्। त्यहाँ सही वा गलत उत्तरहरू छैनन्। कुनै पनि वक्तव्यमा धेरै समय खर्च नगर्नुहोस्।**	मलाई कत्ती पनि (Did not apply to me at all)	मलाई केहि डिग्री/हद सम्म, वा केहि समय भयो(Applied to me to some degree, or some of the time)	मलाई पर्याप्त मात्रामा निकै समयनै भयो(Applied to me to a considerable degree or a good part of time)	मलाई एकदम धेरै वा धेरै समय भयो(Applied to me very much or most of the time)
**१. मैले आफूलाई शक्तिहीन भएको महसुस गरें (I found it hard to wind down)**	○	○	○	○
**२. मैले मेरो मुख सुकेको महसुस गरे (I was aware of dryness of my mouth)**	○	○	○	○
**३. मैले कुनै पनि सकारात्मक भावना अनुभव गर्न सकिनँ I couldn't seem to experience any positive feeling at all)**	○	○	○	○
**४. मैले सास फेर्न कठिनाई अनुभव गरें (उदाहरणका लागि छिटो छिटो श्वासप्रश्वास, वा शारीरिक श्रमको अभावमा सास फेरे जस्तो) (I experienced breathing difficulty (e.g. excessively rapid breathing, breathlessness in the absence of physical exertion)**	○	○	○	○
**५. काम गर्नको लागि पहल गर्न मलाई गाह्रो भयो (I found it difficult to work up the initiative to do things)**	○	○	○	○
**६. म परिस्थितिको अत्यधिक प्रतिक्रिया गर्ने झुकाव राख्दछु म कुनै अवस्थामा आवश्यक भन्दा बढी प्रतिक्रिया दिने भएको छु (I tended to over-react to situations)**	○	○	○	○
**७. मैले कम्पनको अनुभव गरें (उदाहरणका लागि हात काम्ने) (I experienced trembling (e.g. in the hands)**	○	○	○	○
**८. मैले आफूलाई बढी तनाव भएको महसुस गरें (I felt that I was using a lot of nervous energy)**	○	○	○	○
**९. म यस अवस्थामा चिन्तित थिएँ जहाँ म आत्तिएको र आफैंमा एक मूर्ख जस्तो लाग्छ (I was worried about situations in which I might panic and make a fool of myself)**	○	○	○	○
**१०. मलाई अगाडि बढ्न मसँग केही छैन जस्तो लाग्छ /लाग्यो (I felt that I had nothing to look forward to)**	○	○	○	○
**११.मैले आफूलाई रिस उठेको महसुस गरें (I found myself getting agitated)**	○	○	○	○
**१२. मलाई आराम/सुबिस्तासँग बस्न गर्न गाह्रो महसुस भयो (I found it difficult to relax)**	○	○	○	○
**१३. मलाई उदास र निराश महसुस भयो (I felt down-hearted and blue)**	○	○	○	○

१४. म जेसुकै कुरामा असहनशील थिए/भएं जसबाट मलाई जे गरिरहेको थिए त्यसमा लाग्न असमर्थ भएं (I was intolerant of anything that kept me from getting on with what I was doing)	○	○	○	○
१५. मैले आत्तिएको महसुस गरे (I felt I was close to panic)	○	○	○	○
१६.म कुनै कुरामा उत्साहित हुन सकिन (I was unable to become enthusiastic about anything)	○	○	○	○
१७. म एक मान्छे हुँन (मान्छेको रुपमा) धेरै लायक छैन जस्तो मैले महसुस गरे (I felt I wasn't worth much as a person)	○	○	○	○
१८. मैले म बडो संवेदनशील भएको महसुस गरें (I felt that I was rather touchy)	○	○	○	○
१९.शारीरिक श्रम बिना नै म मेरो मुटुको ढड्कन वा कार्यको को बारेमा सचेत थिए (जस्तै हृदयको गति बढेको वा मुटुको ढड्कन नै नभएको महसुस) (I was aware of the action of my heart in the absence of physical exertion (e.g. sense of heart rate increase, heart missing a beat)	○	○	○	○
२०.म बिना कारण डराएको महसुस गरें (I felt scared without any good reason)	○	○	○	○
२१. मैले जीवन अर्थहीन भएको महसुस गरे (I felt that life was meaningless)	○	○	○	○

» G. Substance Use, Diet and Physical Activities

» » 1. Substance Use

के तपाइले धुम्रपान गर्नुहुन्छ ? (धुम्रपानमा चुरोट, बिडी, पाइप, सिगार, खैनी, सुर्ती, जर्दा, पान, हुक्का, चिलिम र तमाखु पर्दछन्),

- ○ गर्दिन
- ○ हाल गर्छु
- ○ पहिला गर्थे

यदी हाल गर्नु हुन्छ भने, के तपाइले हाल एक हप्ता भित्र धुम्रपान गर्नुभएको छ? .

- ○ छ
- ○ छैन

कति पटक धुम्रपान गर्नुभएको छ ?

____

के तपाइले गएको ७ दिन भित्र मदिरा खानु भएको थियो?

- छ
- छैन

कति पटक मदिरा खानु भएको छ ?

» » 2. Diet

सामान्यता एक हप्तामा तपाइ कति पटक फलफुल खानु हुन्छ?

सामान्यता एक हप्तामा तपाइ कति पटक तरकारी खानु हुन्छ?

» » 3. Physical Activties

» » » Vigorous Physical Activities (पछिल्लो सात दिनमा तपाईंले गर्नुभएका सबै कडा शारीरिक गतिविधिहरूका बारे सोच्नुहोस्। जोरदार वा कडा शारीरिक गतिविधि भनेको गतिविधिहरू जुन कठिन शारीरिक प्रयास लिन्छ र तपाईंलाई सास फेर्न गाह्रो बनाउँदछ । केवल ती शारीरिक गतिविधिहरूको बारेमा सोच्नुहोस् जुन तपाईंले एक पटकमा कम्तिमा १० मिनेट गर्नुभयो ।)

पछिल्लो सात दिनमा, तपाईंले जोरदार शारीरिक गतिविधिको कार्य गर्नु भएको छ ? (जस्तै भारी सामान उठाउँने, खन्ने काम, एरोबिक्स, वा द्रुत गतिमा साइकल चलाउने)

- छ
- छैन

यदी छ भने , पछिल्लो सात दिनमा, तपाईंले कत्ति दिन जोरदार शारीरिक गतिविधिको कार्य गर्नुभयो (जस्तै भारी सामान उठाउँने, खन्ने काम, एरोबिक्स, वा द्रुत गतिमा साइकल चलाउने) ?
days per week

ती दिनहरूमा तपाईं सामान्यतया जोडदार शारीरिक गतिविधिहरू गर्न कति समय बिताउनु भयो?
minutes per day

» » » Moderate Physical Activities (पछिल्लो सात दिनमा तपाईंले गर्नुभएका सबै मध्यम शारीरिक गतिविधिहरूका बारे सोच्नुहोस्। मध्यम शारीरिक गतिविधि भनेको गतिविधिहरू जुन मध्यम शारीरिक प्रयास लिन्छ र तपाईंलाई सास फेर्न सामान्य भन्दा केहि गाह्रो बनाउँदछ । केवल ती शारीरिक गतिविधिहरूको बारेमा सोच्नुहोस् जुन तपाईंले एक पटकमा कम्तिमा १० मिनेट गर्नुभयो ।)

पछिल्लो सात दिनमा, तपाईंले मध्यम शारीरिक गतिविधिहरू गर्नु भएको छ ? जस्तै हल्का भार बोक्ने, नियमित गतिमा साइकल चलाउने (Exclude walking)

- छ
- छैन

पछिल्लो सात दिनमा, तपाईंले कति दिन मध्यम शारीरिक गतिविधिहरू गर्नुभयो जस्तै हल्का भार बोक्ने, नियमित गतिमा साइकल चलाउने ?(Exclude walking) Think about all the moderate activities that you did in the last 7 days. Moderate activities refer to activities that take moderate physical effort and make you breathe somewhat harder than normal. Think only about those physical activities that you did for at least 10 minutes at a time.

days per week

ती दिनहरूमा तपाईं सामान्यतया मध्यम शारीरिक गतिविधिहरू गर्न कति समय बिताउनु भयो?

minutes per day

» » » Walking (Think about the time you spent walking in the last 7 days. This includes at work and at home, walking to travel from place to place, and any other walking that you have done solely for recreation, sport, exercise, or leisure.)

पछिल्लो सात दिनमा, तपाईं कम्तिमा १० मिनेट हिंड्नु भएको छ ? (जस्तैः घर बाट काममा हिड्दा, कुनै पनि अन्य पैदल यात्रा जुन तपाईंले केवल मनोरन्जन, खेल, व्यायाम, वा फुर्सतको लागि गर्नुभएको)

- छ
- छैन

पछिल्लो सात दिनमा, कति दिन तपाईं कम्तिमा १० मिनेट हिंड्नुभयो ? (जस्तैः घर बाट काममा हिड्दा, कुनै पनि अन्य पैदल यात्रा जुन तपाईंले केवल मनोरन्जन, खेल, व्यायाम, वा फुर्सतको लागि गर्नुभएको)

days per week

ती दिनहरूमा तपाईं कती समय प्राय: हिँड्न बिताउनु भयो?

minutes per day

तपाईंले कति समय बसेर, ढल्केर अथवा अडेस लगाएर बिताउनुहुन्छ? The last question is about the time you spent sitting on weekdays during the last 7 days. Include time spent at work, at home, while doing course work and during leisure time. This may include time spent sitting at a desk, reading, or sitting or lying down to watch television.

hours per day

What is your vaccination status ?

- ◯ vaccinated
- ◯ Not vaccinated
- ◯ Dont know

If vaccination is presentation, please keep record of the person and inform that to us

के तपाईं अन्त्यमा केहि भन्न चाहानुहुन्छ?

सहभागिताको लागि धन्यबाद
